# Prof. Mulder’s Chemical Counterblast against the Potato as an Article of Diet

**Published:** 1849-02

**Authors:** 


*Professor Mulder's Chemical Counterblast against the Potato as an artical of Diet.*—In a work recently published, Mulder, the learned Professor of Utrecht, has put forth a counterblast against the potato. As an article of diet, he regards it as innutritious, and contends that it is the cause of the moral and physical degeneration of those nations which use it as food! He admits that life may be supported on potatoes alone; but it is not an elastic or healthy life! In fact, the potato fills the stomach with a mass of provender, from which but little healthy nutriment can be extracted. He contends that we shall never see the abuse of spirituous liquors got rid of until potatoes are abolished as a common artile of food, on the principle that a certain amount of stimulus is indispensable, and that therefore the ignorant will have recourse to one that is destructive to them, so long as a salubrious excitement is denied.—*Lond. Med. Gaz.—Med. News.*

